# Blood-Brain Barrier Dysfunction in CNS Disorders and Putative Therapeutic Targets: An Overview

**DOI:** 10.3390/pharmaceutics13111779

**Published:** 2021-10-26

**Authors:** Sabrina Rahman Archie, Abdullah Al Shoyaib, Luca Cucullo

**Affiliations:** 1 Department of Pharmaceutical Sciences, Texas Tech University Health Sciences Center, Amarillo, TX 79106, USA; Sabrina.Archie@ttuhsc.edu (S.R.A.); abdullahal.shoyaib@ttuhsc.edu (A.A.S.); 2 Department of Foundational Medical Studies, Oakland University William Beaumont School of Medicine, Rochester, MI 48309, USA

**Keywords:** biological barriers, tight junctions, endothelial, dysfunction, stroke, viability, therapeutic targets

## Abstract

The blood-brain barrier (BBB) is a fundamental component of the central nervous system (CNS). Its functional and structural integrity is vital to maintain the homeostasis of the brain microenvironment by controlling the passage of substances and regulating the trafficking of immune cells between the blood and the brain. The BBB is primarily composed of highly specialized microvascular endothelial cells. These cells’ special features and physiological properties are acquired and maintained through the concerted effort of hemodynamic and cellular cues from the surrounding environment. This complex multicellular system, comprising endothelial cells, astrocytes, pericytes, and neurons, is known as the neurovascular unit (NVU). The BBB strictly controls the transport of nutrients and metabolites into brain parenchyma through a tightly regulated transport system while limiting the access of potentially harmful substances via efflux transcytosis and metabolic mechanisms. Not surprisingly, a disruption of the BBB has been associated with the onset and/or progression of major neurological disorders. Although the association between disease and BBB disruption is clear, its nature is not always evident, specifically with regard to whether an impaired BBB function results from the pathological condition or whether the BBB damage is the primary pathogenic factor prodromal to the onset of the disease. In either case, repairing the barrier could be a viable option for treating and/or reducing the effects of CNS disorders. In this review, we describe the fundamental structure and function of the BBB in both healthy and altered/diseased conditions. Additionally, we provide an overview of the potential therapeutic targets that could be leveraged to restore the integrity of the BBB concomitant to the treatment of these brain disorders.

## 1. Introduction

Biological barriers perform a significant role in maintaining the integrity and function of many vertebrate organs. Intercellular protein complexes of the plasma membrane form paracellular diffusion barriers that separate internal and external fluid compartments, which is a crucial process for the development and function of all organs [1]. Different biological barriers are present in different body organs such as the skin, intestine, kidney, reproductive system, lung, liver, mouth mucosa, and central nervous system (CNS). The CNS is protected from the external environment by three biological barriers at three interfaces. These are the blood-brain barrier (BBB), the blood–CSF barrier (BCB), and the arachnoid barrier [2]. The BBB is formed by the endothelial cells lining cerebral microvessels and separates the blood from brain interstitial fluid. The choroid plexus epithelium is positioned between the blood and ventricular cerebrospinal fluid (CSF) and forms the blood-CSF barrier. The arachnoid barriers are formed by epithelium positioned between the blood and subarachnoid CSF. These three barrier layers participate in limiting and regulating molecular exchange at the interfaces between the blood and the neural tissue or its fluid spaces. Among these CNS barriers, the BBB exerts the tightest control over the surrounding brain microenvironment. More specifically, the BBB provides barrier functions at three different levels.

The first is a physical barrier that blocks the paracellular pathway of polar substances (including ions) between adjacent endothelial cells. Tight junctional proteins form this barrier through homotypic binding with their homologous counterpart on the adjacent endothelial cells. Different efflux transporters provide the second barrier, with a wide range of affinity for lipophilic substances. These include a P-glycoprotein (P-gp), breast cancer resistant protein (BCRP), and multidrug resistance-associated proteins (MRPs) [2]. The third is a multi-enzymatic barrier that provides the BBB with a certain degree of drug metabolism capabilities [3]. The brain capillary contains a wide range of neurotransmitter-metabolizing enzymes, including cholinesterases, GABA transaminase, aminopeptidases, and endopeptidases, as well as several drug- and toxin-metabolizing enzymes [4]. Thus, the enzymatic blood-brain barrier protects the brain from circulating neurotransmitters as well as from many toxins.

**Figure 1:**
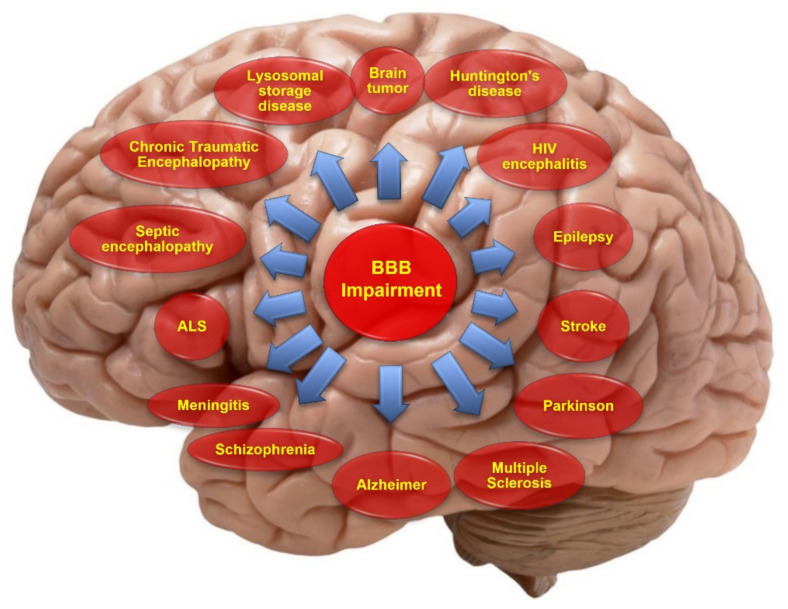
Schematic illustration summarizing some of the major neurological disorders associated with impairment of the BBB. Note that in some cases, there is a clear, direct causative association where impairment of the BBB is the major prodromal factor for the onset and/or progression of the CNS disorder (e.g., post-ischemic brain injury). In other instances, whether the BBB damage is the causative factor or a derivate effect of the brain disorder further impacting the disease at a later stage is less clear (e.g., Epilepsy).

Considering the pivotal role the BBB plays in maintaining brain homeostasis and protecting the CNS, it is understandable that dysfunctions of the BBB can be prodromal to the onset of neurological and spine disorders and/or the worsening of brain disorders. Therefore, it is not surprising that an impairment of the BBB has been associated with severe and detrimental outcomes in the context of many neurological disorders (see Figure 1) [2,5,6]. Since impairment of the BBB is closely linked to many CNS disorders, therapeutic targets that aim to restore the BBB’s viability could be a feasible method to reduce the burden of diseases and improve the outcome for a patient. Hence, this review highlights the pathological conditions associated with dysfunction of the BBB and the therapeutic targets currently exploited to promote BBB restoration.

## 2. The Function of the BBB

The overall components of the BBB participate in maintaining a stable microenvironment which is important for sustaining complex neural functions and protecting the CNS. The BBB contains different ion channels and transporters, which help maintain the ionic balance for synaptic signaling activity. Potassium (K^+^), magnesium (Mg^2+^) and calcium (Ca^2+^) ions are regulated at the BBB and BCSFB [2,5,6]. For instance, the concentration of potassium (K^+^) in mammalian plasma is around 4.5 mM. In contrast, in CSF and ISF, it is approximately 2.5–2.9 mM, and it is not affected by external factors such as exercise, meal intake, pathological state, or experimental condition [7,8].

Moreover, the BBB plays a pivotal role in sustaining brain nutrition through a plethora of mechanisms. The BBB has a low passive permeability for water-soluble nutrients and metabolites essential for the nervous tissue. On the other hand, specific transporters present in the BBB allow for the transportation of other essential substances, for instance, glucose and amino acids that cannot pass through the BBB. Expression of these selective and region-specific (luminal and abluminal surfaces of the ECs) transporters ensures BBB endothelium polarization [3,9]. Further, the BBB plays a significant role in regulating the neurotransmitter in CNS by keeping the central and peripheral neurotransmitter pool separated. Uncontrolled release of neurotransmitters such as glutamate into the brain CSF during hypoxia in ischemic stroke conditions may cause severe and permanent neurotoxic damage [3,10].

Additionally, the movement of macromolecules between blood and brain parenchyma is controlled and maintained by the BBB. CSF contains a lower concentration of proteins than in plasma as the BBB prevents the entry of macromolecules into the CNS. Different plasma proteins, such as albumin, prothrombin, and plasminogen, may cause cellular apoptosis, damaging nerve tissues [11,12,13]. Factor Xa converts prothrombin to thrombin, and the tissue plasminogen activator converts plasminogen to plasmin. Both factor Xa and the tissue plasminogen activator are present in the brain. Leakage of these large macromolecular proteins into the brain through a disrupted BBB may result in severe pathological consequences, including seizures, glial cell activation and division, scarring, and cell death [13]. The BBB also serves as a shield to protect the CNS from various toxins circulating in the blood. These toxic molecules can be endogenous proteins, metabolites, or xenobiotics obtained through diet or environmental pollutants. If they gain entry into the brain, they may compromise neuronal activity and/or promote cell death [2].

## 3. Structure of the Blood-Brain Barrier: An Overview

**Figure 2:**
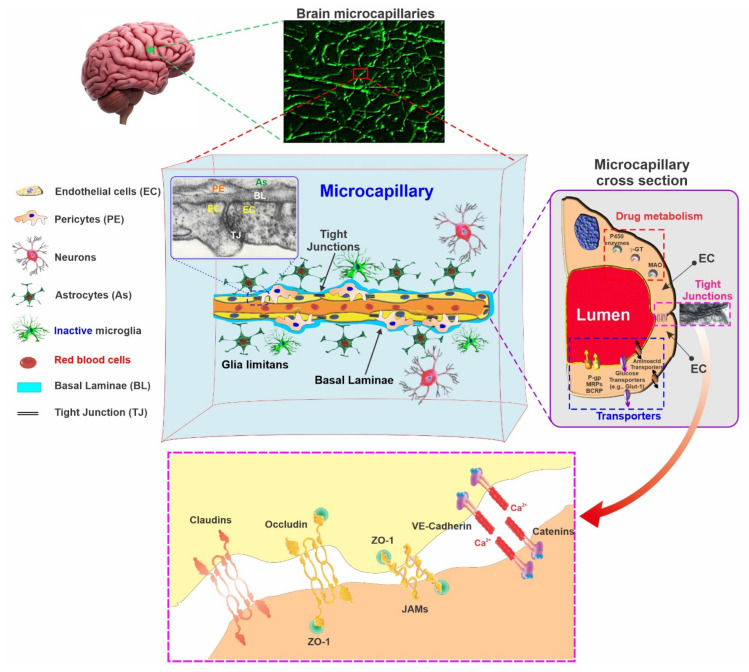
Schematic illustration of the BBB anatomy. A cross-section of a brain microcapillary segment depicting the innermost luminal compartment composed of a uniform layer of a tightly packed endothelial cell (EC) surrounded by an additional envelope of pericytes (embedded within the basal membrane and astrocytic foot processes which tightly ensheaths the brain microcapillary. The movement of substances across the BBB endothelium is controlled by a multimodal barrier that includes tight junctions (gating barrier to paracellular diffusion of polar molecules); efflux transporters (P-gp, MRPs, BCRP, etc.) with high affinity for lipophilic substances such as cytochrome P450 enzymes, MAO, etc. (metabolic/enzymatic barrier).

The development of the BBB begins during the fetal stage, and it is well constructed by the point of birth, especially for macromolecules and proteins [14,15,16,17,18,19,20,21]. At the cellular level, the BBB consists of microvascular endothelial cells (EC) lining the luminal walls of brain microvessels alongside closely associated pericytes embedded within the basal membrane and surrounded by astrocytic end-feet processes (see Figure 2) that support EC’s phenotypic differentiation and the maintenance of BBB features [2,3]. The microcapillary endothelium is characterized by the expression of tight junctions (TJs), a lack of fenestrations, negligible pinocytotic trafficking, and distinct asymmetrical distribution patterns of the transmembrane transporters, which provides the BBB endothelium a cellular polarization. The presence of TJs such as occludin (OCLN), claudin-5 (CLN-5), and junctional adhesion molecules (JAMs) are important for the BBB. These interendothelial junctions form a diffusion barrier that selectively prevents most blood-borne and xenobiotic hydrophilic substances from entering the brain through paracellular routes, protecting it from any undesired systemic and external influences [2,22]. Occludin and claudins are associated with cytoplasmic scaffolding and regulatory proteins called zona occludens (ZO-1, ZO-2, ZO-3) and cingulin, which anchor the TJs to the actin cytoskeleton, provide signaling functions, and guide the TJs in membrane distribution. In addition to TJs, the interendothelial space also features junctional complexes, including the adherens junction (AJs) and proteins such as cadherins. VE-cadherin (also known as Cadherin-5 or CD144) plays a particularly important role in the maintenance of cell-cell adhesion, contact inhibition, cytoskeleton remodeling, intracellular signaling, BBB permeability (via the modulation of CLN-5 expression [23]), and angiogenesis) [24]. Therefore, the loss of the AJ can lead to a loss of BBB integrity and its increased permeability [23,25]. However, the function and properties of the barrier do not entirely depend on the presence and expression of claudins and/or OCLN but rather on the organization, distribution, and interactions of these TJs with their homotypic counterpart on the adjacent cells [26].

Considering the intrinsic properties of the TJs physical barrier, the transport of water-soluble nutrients (such as D-glucose, monocarboxylic acids, and essential amino acids) from peripheral circulation into the brain parenchyma depends upon specific carrier-mediated transport systems with which the BBB endothelium is enriched, as summarized below. Although BBB properties are a characteristic feature of the brain microvascular endothelium, they are not intrinsic properties of the BBB endothelium. Rather, they are primarily acquired through an endothelial interaction with the surrounding environment during a process known as barriergenesis. Cellular cues derived from mural cells, immune cells, glial cells, and neural cells, all of which are part of the neurovascular unit (NVU) [27] during the various phases of brain development, are essential for the differentiation of the vascular endothelium into a BBB phenotype.

### 3.1. Endothelial Cells (ECs)

The brain microvascular endothelium is distinct from other tissue ECs, allowing them to tightly control the passage of ions, molecules, and cells between the blood and the brain. ECs of the CNS microcapillaries are joined by tight junctions (TJs), that restrict the paracellular transport of polar solutes [28,29,30]. Compared with the peripheral vasculature, the BBB endothelium exhibits an extremely low transcytosis and vesicle-mediated transcellular passage of solutes [31]. In addition, these microvascular endothelial cells present a regional polarization of transport mechanisms (both qualitative and quantitative) between the luminal and the basolateral side of the cell membrane so as to better control the influx and efflux of substances to and from the brain [32,33]. Transcytosis is an apical-to-basolateral vesicular-dependent intracellular transport mechanism and one of the key features of BBB. Transcytosis in brain endothelial cells is partially responsible for transporting several large molecules, including fatty acids and transferrin, across the BBB [34,35]. Transcytosis activity is higher during the early development period in the brain endothelial cells; however, it becomes dormant during the course of BBB maturation. Therefore, the upregulation of transcytosis (increased intracellular vesicles in the brain endothelial cells) is considered an early and precise indicator of BBB disruption [36,37]. Although it was previously assumed that changes in tight junction proteins primarily facilitated BBB permeability, recent evidence obtained from mice and zebrafish suggests that transcytosis is equally important in the regulation of BBB integrity [34].

Efflux transporters are primarily localized to the luminal surface of the BBB endothelial cells. They include the P-glycoprotein (P-gp), breast cancer resistance protein (BCRP), and multidrug resistance associate proteins (MRPs) such as MRP1, MRP3, MRP4, and MRP6. Through the use of ATP hydrolysis, these transporters manage to drive their potentially harmful amphipathic and hydrophobic substrates across the ECs membrane back into the bloodstream against a concentration gradient, thus preventing them from entering into the brain [38,39]. These transporters function in parallel with various enzymes such as cytochrome P450s, monoaminoxidase (MAO), cholinesterases, catechol *O*-methyl transferase (COMT), endopeptidases, aminopeptidases, etc., that further increase the ability of BBB to remove/neutralize potentially harmful substances [3,40,41,42,43]. In contrast, other specialized transporters (that are mostly ATP independent), belonging to the solute carrier (SLC) superfamily, mediate the movement of small anionic and cationic molecules as well as nucleosides and peptides across the BBB, but also remove waste products from the CNS into the blood [44]. A brief overview of these transporters is presented here, although the reader is referred elsewhere for a more detailed and comprehensive description [45].

Moreover, the BBB endothelium has a higher amount of mitochondria compared to other vascular ECs. This is crucial to produce the necessary for ATP to support the metabolic work capability of the BBB and provide the ion gradient required for some of the transport functions [46]. Additionally, BBB ECs express very low amounts of leukocyte adhesion molecules (LAMs including E- and P-selectins) required for leukocyte entrance into the CNS across the endothelium [47,48], thus limiting the movement of immune cells into the brain [49,50,51], which is a de facto an immune-privileged organ. However, an elevated expression of LAMs was observed in the setting of neuroinflammatory diseases [47,49,51,52]. For instance, the infiltration of T and B lymphocytes, neutrophils, and macrophages was observed at multiple sclerosis (MS) active injury sites. Moreover, the infiltration of neutrophils, macrophages, and lymphocytes was reported in stroke [27,53].

### 3.2. The Basement Membrane

The endothelial vascular tube is surrounded by the following two types of basement membranes (BM): the inner vascular basement membrane and the outer parenchymal basement membrane. The inner vascular BM is composed of an extracellular matrix that is secreted by endothelial cells and pericytes. On the contrary, the outer parenchymal BM is secreted by astrocytic processes. These basement membranes are mainly comprised of laminin, agrin, type IV collagens, nidogen, heparin sulfate proteoglycans, and other glycoproteins. The vascular and parenchymal BM possess different isoforms of laminin. The vascular BM is composed of laminin α4 and laminin α5, whereas the parenchymal BM is comprised of laminin α1 and laminin α2 [54,55]. These basement membranes perform a pivotal role in many signaling processes and act as an additional barrier prior to access to the neural tissue. It is well acknowledged that matrix metalloproteinase enzymes (MMPs) disrupt the basement membranes, resulting in BBB dysfunction at the onset of different neurological disorders [27].

### 3.3. Astrocytes

Astrocytes are the most abundant cells in CNS and are responsible for the operation of different physiological and biological functions, including, but not limited to, neural parenchyma compartmentalization, pH regulation, ionic homeostasis, neurotransmitter uptake, and processing and signal mediation from neurons to vasculatures [56]. Interestingly, the glial/neuron ratio increases dramatically with brain complexity and volume (3). At the level of arterioles and venules, astrocytes contribute to the cerebral blood flow regulation through the release of vasoactive substances, thus modulating the vascular tone. At the brain microvascular level, astrocytes promote the differentiation of cerebrovascular endothelial cells to a BBB phenotype and contribute to the modulation of mature BBB functions and activity such as the induction and maintenance of tight junctions [2,57,58]. In vitro studies have also revealed that markers related to the development of a functional BBB, such as transferrin receptor, P-glycoprotein, gamma-GTP, etc., are typically upregulated in endothelial cells when co-cultured with astrocytes [58,59].

Recently, studies have demonstrated the crucial role of astrocytic endfeet in brain metabolism [9]. The communication between the astrocytes and the underlying microcapillaries occurs via the astrocytic endfeet, which ensheath the vascular tubes. These endfeets contain various proteins, including dystroglycan, dystrophin, aquaporin 4 (Aqp4), and the dystroglycan–dystrophin complex, which connects the endfeet cytoskeleton to the basement membrane through its binding with agrin [60,61]. Aqp4 is coordinated by this connection, which assists in maintaining cerebral water and ion homeostasis [27,62] as well as neurotransmitter regulation [63]. In contrast, activated astrocytes can secrete pro-inflammatory cytokines acting as inflammatory modulators and neurotoxins, causing neuronal damage [64,65].

In vivo studies have shown that the BBB is already experiencing development before the astrocytes envelop the brain capillaries; it has therefore been concluded that while astrocytes may not perform a significant role in the initial setting of BBB development, they enact a crucial role in the modulation and maintenance of BBB integrity post-formation [27,66].

### 3.4. Mural Cells and Pericytes

The vascular smooth muscle cells surrounding the large vessels and pericytes are known as mural cells. The pericytes are essential components of the brain capillary located on the abluminal surface of the microvascular endothelial tube and are embedded in the vascular BM [67]. They share a BM with endothelial cells, displaying a direct synaptic-like peg-socket focal contact with endothelium through N-cadherin and connexins [68]. CNS vasculature contains the greatest number of pericytes compared to other tissues with an approximate endothelial and its pericyte ratio is between 1:1 and 3:1 while the ratio is 100:1 for the muscle [69].

Pericytes are associated with different functions, including but not limited to maintaining BBB integrity, angiogenesis, microvascular stability, the regulation of the capillary diameter and cerebral blood flow, the deposition of the extracellular matrix, the regulation of immune cell filtration, and the removal of toxic metabolites [68,70,71,72,73]. Additionally, pericytes are crucial for regulating the formation of BBB during its development (Barriergenesis) and maintaining its integrity and function during adult and senior stages [66,74]. Moreover, other studies have demonstrated that pericytes perform an important role in cerebral autoregulation by expressing receptors for vascular mediators such as, angiotensin I [75], catecholamine [76], vasopressin vasoactive intestinal peptides [77], endothelin-1 [78], and vasopressin [79]. Different molecular identifiers, including PDGFR-β, NG2, Anpep (CD13), desmin, Rgs5, Abcc9, Kcnj8, Dlk, and Zic1 Immune Cells have been used to distinguish pericytes [68,80,81,82], although none of these are perfect identifiers of this cell type. It is challenging to determine pericytes’ precise involvement and functional contribution due to the lack of specific classification parameters (to define the exact type of pericyte) and corresponding specific markers [27].

### 3.5. Immune Cells

The blood vessels in the CNS interact with different immune cells, both in the CNS and in the blood. Perivascular macrophages and microglial cells are the principal immune cells within the CNS. Macrophages are monocyte lineage cells derived from blood-borne progenitors and reside on the vascular tube’s abluminal side [83,84]. These cells can phagocyte cellular debris and act as the first line of innate immunity [27]. Multiple studies have demonstrated that these cells can cross the BBB, and 80% of them are replaced within three months [85,86,87].

Microglial cells reside in CNS parenchymal immune cells that are derived from progenitors in the yolk sac and enter the brain during embryonic development [88]. These cells regulate neuronal development, the innate immune response, and wound healing and act as antigen-presenting cells in adaptive immunity [89,90]. Additionally, the interaction of blood-borne immune cells (neutrophils, T cells, and macrophages) with CNS vessels is also assumed to be integral for the maintenance of BBB integrity as they are capable of increasing vascular permeability by releasing reactive oxygen species once they are activated in response to an injury, disease condition or infection [91,92]. Therefore, it is crucial to identify the underlying mechanism behind the activation of both immune cells and the BBB and the interaction between the two to better understand the mechanisms that cause BBB disruption across different neurological diseases [27].

## 4. Adrenergic System and BBB

Several studies performed during the 1990s demonstrated the influence of the adrenergic system on BBB permeability. An increased BBB permeability was observed due to α-adrenoceptor activation either by the intracerebroventricular administration of agonists [93] or through the electrical stimulation of the locus coeruleus [94]. Moreover, the permeability of the BBB was facilitated by the blockade of the β-adrenoceptor. As a result of the α-adrenoceptor blockade and β-adrenoceptor stimulation, decreased BBB permeability became evident in various studies [95,96]. In all of these studies, alterations in the BBB permeability were accompanied by changes in pinocytotic activity in brain microvessel endothelial cells; however, the morphology of TJs remained unchanged [97].

## 5. BBB Dysfunction in CNS Disorders

The functional alterations of structural and cellular components in the BBB are responsible for BBB disruptions. These alterations may include changes that occur in tight junction expression, their distribution, and the local microenvironment that could be conducive to the opening of TJs, transport systems, enzymes, and the disruption of the basement membrane, which may ultimately lead to serum components and immune cell infiltration into the brain parenchyma, disrupt the CNS homeostasis and damage the surrounding brain tissues. Several studies have demonstrated that the disruption of the BBB is related to the onset and progression of various neurological and cerebrovascular diseases, including stroke, traumatic brain injury, brain tumor, multiple sclerosis, Alzheimer’s and Parkinson’s disease, epilepsy, edema, glaucoma, and amyotrophic lateral sclerosis. However, whether the disease conditions result from BBB impairment or BBB disruption occurs due to the disease pathology is still somewhat in dispute (for instance, in epilepsy). However, barrier disruption is often observed and can contribute to and exacerbate the developing pathology (see Table 1) [98]. We provide a summary overview of major CNS disorders and the implication of BBB impairments below.

### 5.1. Stroke

Stroke is the leading cause of permanent disability and is associated with various comorbidities such as hypertension and hyperglycemia [119]. Around 86% of stroke incidents are ischemic and are a result of the interruption of the blood and oxygen supply to a particular brain region [90], leading to a series of interrelated pathophysiological cascades that include but are not limited to BBB impairments [120,121]. The BBB’s disruption appears to start immediately after vessel occlusion and continues after the stroke event for an extended period [122]. However, it is not yet clear if the BBB disruption is the cause or the consequence of the post-stroke injury [123]. It has been observed from different experimental studies that hypoxia-ischemia conditions can affect the BBB by disrupting the TJs and damaging endothelial cells, resulting in increased permeability [124]. The TJs ensure a low paracellular permeability, ultimately preventing the occurrence of unwanted ion fluxes and paracellular diffusion across the BBB [125]. However, during ischemic stroke, the degradation of TJs occurs in a multistep and time-dependent way, comprising several signaling mechanisms [126]. In healthy conditions, the stability of TJ is maintained by anchoring the tight and adherens junctions (AJs; e.g., cadherin) to the actin cytoskeleton through tight junction-associated proteins such as ZO-1, ZO-2 and ZO-3 that act as linkers. The actin-myosin cytoskeleton is distributed in the form of short filaments and diffuse monomers between the endothelial cells. However, when subjected to hypoxic stress, the actin filaments polymerize into linear stress fibers, and the actin-myosin cytoskeleton contracts via myosin light chain phosphorylation, resulting in an increased cytoskeletal tension, the deterioration of the junction seals, and an increased BBB permeability [127,128,129]. Decreased expression levels in TJ transmembrane proteins (occludin, claudins, zona occludens, and junction adhesion molecules) has also been observed in stroke brains [125,126].

Pericytes, which constitute a functional component of NVU, closely interact with the capillary and venule endothelial cells via paracrine signaling and physical contact [130]. Pericyte plays a critical role in BBB maintenance due to its contractile, inductive, structural, and regulatory properties [131,132]. During an ischemic stroke, blood vessel constriction and a loss of pericytes occur, leading to a decreased cerebral blood flow and the loss of BBB integrity. It has been reported that during the hypoxic phase of an ischemic stroke, pericytes migrate from their original microvascular location, thereby impairing the viability of the BBB [131]. Moreover, an in vivo study in mice stroke models reported that the pericyte-derived vascular endothelial growth factor (VEGF) promotes the loss of BBB integrity in favor of angiogenesis following a stroke event [133]. VEGF is a crucial proangiogenic factor that stimulates endothelial cell proliferation, and its migration has been found to produce beneficial effects when administered before or after a stroke occurrence [134,135,136]. However, if VEGF is administered during the post-stroke acute phase, it can promote a leakage in BBB and cause a cerebral hemorrhage, resulting in an increased infarct volume [136].

Astrocytes, another crucial component of the NVU, participate in the maintenance of the BBB. However, they can also promote BBB disruption during an ischemic stroke. Astrocytes perform a dual role, depending on the phase of ischemia. During the acute phase, astrocytes are activated and secrete proinflammatory cytokines, inhibiting axonal generation, thus producing harmful effects. In contrast, astrocytes can perform a protective role during the chronic phase by participating in neurite sprouting, synapse formation, neurotrophic factor secretion, and rebuilding the BBB [137,138].

These events may be mediated by the release of soluble factors, such as cytokines, vascular endothelial growth factor (VEGF), and nitric oxide (NO). Higher levels of pro-inflammatory cytokines, including IL-1β, and TNF-α have been reported in animal brains after focal and global ischemia [139] and in the CSF of stroke patients [140]. It has also been observed, in vitro, that ischemic conditions can induce the secretion of IL-8 and monocyte chemoattractant protein-1 (MCP-1) in astrocyte and endothelial cells [141]. Another study has demonstrated that human astrocytes release inflammatory mediators under hypoxia, resulting in the upregulation of IL-8, ICAM-1, E-selectin, IL-1 β, TNF-α, and of MCP-1 genes in human cerebrovascular endothelial cells. The elevated level of cytokines upregulates endothelial and neutrophil adhesion molecules, resulting in the transmigration of leukocytes across the endothelium and the BBB. The recruitment of leukocytes is characterized by increased phosphotyrosine staining, decreased TJs proteins, and the redistributed AJs protein (vinculin), indicating BBB disruption [142].

Additionally, Mark KS et al. reported that under hypoxic conditions, sucrose permeability across primary bovine brain microvessel endothelial cells increased by more than 2.5 folds along with elevated expression of actin and altered the distribution of OCLN, ZO-1, and ZO-2 proteins [143]. Collectively, these experimental findings suggest that hypoxia-ischemia conditions can trigger the disruption of TJs and the loss of BBB integrity through a cascade of events involving VEGF, cytokines, and NO.

### 5.2. Multiple Sclerosis (MS)

Multiple sclerosis is an autoimmune disease in which reactive T cells interact with the antigen presented by macrophages- or microglia-expressing HLA-DR2a and HLADR2b. It leads to the destruction of the myelin sheath and the underlying axons [144]. NO, and various cytokines (interferon-γ, TNF-α, and IL-3), secreted by activated macrophages, damage oligodendrocytes and interfere with myelination and myelin gene expression [145,146]. Moreover, elevated amounts of reactive oxygen species (ROS) have been detected in MS lesions, leading to brain damage and contributing to several mechanisms underlying the pathogenesis of MS lesions [147]. As radiographic and histopathological evidence suggests, BBB disruption is one of the initial critical steps in multiple sclerosis. Radiographic analyses displayed Gd enhancing lesions; markers of BBB disruption are related to the active inflammation in the lesions and are considered an important diagnostic marker of MS [148].

Additionally, histopathological studies have revealed the origination of the myelin breakdown around parenchymal blood vessels [149]. Since BBB disruption creates a gateway for the entrance of inflammatory infiltrates into the perivascular space, it is hypothesized that a loss of BBB integrity could be one of the initial critical events in the lesion formation. This hypothesis is supported by evidence, such as the deposition of fibrinogen (a marker of endothelial permeability) [150], followed by a high infiltration of T cells in the demyelinated foci. TJs abnormalities, including the loss of claudin-3 [151,152], were also observed in the relapsing-remitting and progressive stages of MS, suggesting the opening of paracellular routes [148]. Furthermore, the failure of upregulating AQP4 and the retraction of astrocytic end-feet from the glia limitans [148] and the degradation of the basement membrane protein laminin [123] were both observed in MS, further inducing the loss of BBB viability.

### 5.3. Amyotrophic Lateral Sclerosis (ALS)

Amyotrophic lateral sclerosis (ALS) is a fatal motor neuron disorder characterized by the progressive loss of the upper and lower motor neurons (LMNs) at the spinal or bulbar level [153]. It has been reported, in different studies, that the disruption of the BBB is associated with the loss of motor neurons, neuroinflammation, and motor impairment [154,155,156]. Oxidative stress performs a crucial role in the degeneration and dysfunction of motor neurons and astrocytes [157]. ROS generation in motor neurons, which results from excitotoxic activation, can induce the oxidative damage of glutamate transport in the surrounding astrocytes, resulting in increased excitatory stress, thereby promoting an ALS development [158,159]. Aqp4 and inward rectifying potassium channels (Kir) are essential for maintaining functional BBB astrocyte lining. In the ALS model, the ability of astrocytes to maintain the homeostasis of the surrounding environment is disrupted, and this imbalanced homeostasis negatively impacts the BBB viability, promotes neuronal dysfunction, and ultimately, neuronal cell death [159,160]. *Several in-vivo studies* have demonstrated the breakdown of BBB in a SOD1-G93A animal model for ALS [161,162,163,164,165]. This transgenic mouse model expresses the human SOD1 corresponding to the G93A mutation under the control of the cistronic human SOD1 promotor. Mutations in this gene have been linked to the onset of familial ALS (or Lou Gehrig’s disease), whereby the animals develop paralysis in one or more limbs within a few weeks of age.

Moreover, BBB abnormalities were also discovered in a postmortem study of an ALS patient [166]. Miyazaki et al. reported that MMP-9 activation in ALS patients and ALS animal models resulted in BBB damage prior to motor neuron degeneration. Additionally, there was a lack of association between the PCAM-1-positive endothelium and GFAP-positive astrocyte foot processes in patients and in vivo [154]. A direct correlation was observed between the CSF homocysteine increment and BBB disruption in ALS patients [167]. Another study also reported the astrocytic downregulation of the morphogenic protein sonic hedgehog, which resulted in an interleukin-1β mediated disruption of the BBB [168]. The same study also reported that the IL-1β mediated the upregulation of CCL2, CCL20, and CXCL2, which are pro-inflammatory chemokines that can facilitate immune cell migration, leading to BBB disruption and neuroinflammation. The involvement of TARDBP (a gene encoding for a protein called transactive response DNA binding protein 43 kDa—TDP-43, which regulates gene transcription) and angiopoietin (ANG) genes mutation in poor BBB integrity and neuroinflammation in ALS patients has also been proposed in another study [169]. Endothelial damage and/or impaired endothelium repair have also been proposed as causative factors for ALS onset [170].

### 5.4. Traumatic Brain Injury (TBI)

Traumatic brain injury or TBI is caused by the impact of direct or indirect external mechanical force to the brain, for instance, motor vehicle accidents, falls, assaults, sports-related incidents, etc. [171]. Almost 2.5 million people in the US require emergency care each year, and more than 5.3 million people live with a long-term disability caused by TBI [172,173,174]. The pathophysiology of TBI can be divided into two stages: primary or immediate injury and secondary or delayed injury [175,176]. The primary trauma includes acute pathological changes such as a shearing injury, hematomas, and contusions. The secondary injuries include oxidative stress, inflammation, cerebral edema, excitotoxicity, altered vascular permeability, altered calcium homeostasis, and BBB disruption [171]. Among these abovementioned pathophysiological consequences, disruption of the BBB as mediated by inflammation plays a significant role in the progression of brain injury and long-term neurological deficits associated with TBI [177]. The disruption of BBB is one of the notable pathophysiological features of TBI as related to neuroinflammatory events, which may result in brain edema and cell death. It has been observed that during or post-TBI, astrocytes and microglia can rapidly respond to injury through increased levels of multiple biological effects, which may also affect BBB function [178]. BBB integrity and low paracellular permeability are maintained through the presence and binding of inter-endothelial TJs between adjacent cells. TBI can cause endothelial cells to become damaged by disrupting blood flow, altering tight junction protein expression and the basal membrane, thus disrupting BBB integrity and increasing the paracellular permeability of the barrier [171,179]. Studies have shown that BBB disruption after TBI triggers leukocyte recruitment, inflammatory cell migration, proinflammatory cytokines, and ROS release. The generation of ROS related to TBI can further damage the BBB and mechanical trauma by promoting lipid peroxidation, protein backbone fragmentation, and DNA damage if it remains unchecked [171]. BBB disruption after TBI also stimulates the activation of the coagulation cascade, leading to the formation of intravascular blood clots and subsequent ischemia [175].

Alteration of the BBB following TBI occurs via two steps; the first step occurs within 4–6 h of tissue injury, and the second step occurs three days post-injury, affecting the cortex and the ipsilateral hippocampus [180]. Habgood et al. reported the passage of small and large molecules inside the brain after TBI, indicating the breakdown of the BBB. However, the loss of BBB integrity was temporary, as evidenced by the restoration of the BBB restriction of large and small molecules within 4–5 h and five days post-injury, respectively [152]. However, the findings of this study contrast with those of another group of researchers, indicating that restoring the BBB may require a significantly longer period of up to several years [181]. A recent study has demonstrated several inflammatory mechanisms behind BBB breakdown in mild traumatic brain injury (mTBI or concussion, the most common type of TBI) and hypertension [182]. Oxidative stress has been identified as the main cause of BBB impairment in the sub-acute stages of blast-induced traumatic brain injury (bTBI). Moreover, MMP activation after bTBI can also lead to the oxidative stress-mediated loss of BBB integrity caused by NADPH oxidase [183]. VEGF, MMP, NO, glutamate, and endothelin-1 are specific promoters in the loss of BBB integrity that have been associated with post-TBI astrocyte activation [184]. CSF/serum albumin ratio [185], TJs proteins [186,187], S100β [188,189], and plasma-soluble prion protein (PrPc) [190,191] are, conversely, potential biomarkers that have been associated with BBB disruption.

As suggested by a recent study, chronic traumatic encephalopathy (CTE) is also a neurodegenerative disorder related to repeated mTBIs, underlying the association between CTE development and concussive injuries in athletes and military personnel. However, the underlying molecular pathobiology of CTE is not well understood [192]. Markedly discontinuous or the absence of CLN-5, ZO-1, and BBB-associated tight junction components were observed in the regions of the perivascular p-Tau deposition, alongside immunohistochemical evidence of a damaged BBB [192]. Moreover, BBB disruption in the regions of the perivascular p-τ deposit has been reported in CTE and schizophrenia-diagnosed professional boxers. This p-τ deposition resulted in the loss of CLN-5 and increased extravasation of endogenous blood components such as fibrinogen and IgG [193]. Furthermore, the correlation between caspase-3-cleaved tau accumulation and the upregulation of cleaved-caspase-3 following chronic TBI suggests the involvement of apoptosis and neuroinflammation in the delated disruption of BBB following TBI [194].

### 5.5. Alzheimer’s Disease (AD)

Alzheimer’s disease (AD) is a progressive neurodegenerative disorder characterized by memory impairment [195]. Amyloid β (Aβ) is one of the major constituents of the amyloid plaque found in brain regions of patients with Alzheimer’s disease. The impairment of the BBB has been correlated with the pathogenesis of AD, where elevated levels of Aβ deposition and accumulation negatively affect the barrier integrity [195]. However, various studies have also demonstrated that BBB dysfunction plays a crucial role in generating Aβ [195,196,197] by activating β-secretase and γ-secretase [196,198]. At the BBB level, the receptor for advanced glycation end products (RAGE) is considered the prominent transporter of beta-amyloid into the brain from the systemic circulation.

In contrast, the low-density lipoprotein receptor-related protein (LRP)-1 carries the beta-amyloid in the opposite direction and out of the brain. A study of elderly human control and the AD hippocampi revealed the upregulation of RAGE receptors at the microvascular level while LRP-1 expression was downregulated in AD compared to the controls. The opposite pattern of expression was observed at the neuronal level [199]. The results from this study strongly support the proposition that changes in the relative distribution of RAGE and LRP-1 receptors affecting the brain microvasculature and neurons are a prodromal feature of AD. This study also suggests that a significant proportion of the amyloid accumulating within the brain is likely to originate from systemic circulation.

Additionally, changes and dysfunction in the BBB structural components, including pericytes, glial cells, vascular endothelial cells, the basement membrane protein (argin), and TJs, have been associated with an increased risk of AD [199]. Additional studies have also demonstrated that AD is associated with a decreased level of the glucose transporter, GLUT-1 [200], and p-glycoprotein [201]. Other features of AD, such as neuroinflammation and oxidative stress, both promoting BBB dysfunction, can reinforce the pathogenic cycle, thereby associating BBB alteration with the onset and progression of the disease [202,203,204]. However, additional studies are necessary to better dissect the pathogenic cascade leading to the onset of AD and to determine whether and to what measure BBB dysfunction acts as a causative factor or a derived effect that further contributes to the progressive worsening of the disease. For more detailed and extensive information on the subject, we refer the readers to recently published literature [205,206].

### 5.6. Parkinson’s Disease (PD)

Parkinson’s disease (PD) is a cognitive disorder that causes movement dysfunction. It is associated with multiple pathologic characteristics, including the formation of certain proteinaceous inclusions inside neurons, known as Lewy Bodies, and the loss of dopaminergic neurons in the Substantia Nigra pars compacta [207]. Although PD is related to multiple gene mutations, they are not isolated factors in promoting the onset of PD. Furthermore, the disease is thought to be related to a range of polygenetic and environmental cues. For example, 1-methyl-4-phenyl-1,2,3,6-tetrahydropyridine (MPTP) is a lipophilic compound and crosses the BBB easily, that has been found to induce symptoms and the pathology of PD in the case of in vivo models [208]. Furthermore, a reduced P-gp function at the BBB has been observed in PD patients suggesting that a dysfunctional BBB may act as a causative mechanism in the onset of the disease [209,210]. Additional observations have revealed systemic vascular inflammation in PD patients [211], which may also harm the BBB; however, evidence of increased BBB permeability in PD patients is still lacking.

### 5.7. Huntington’s Disease (HD)

Huntington’s disease (HD) is an autosomal dominant inherited invariably fatal disorder that results from the expansion of glutamine residues in the HTT gene encoding for a protein called huntingtin (htt) as a result of a mutation in CAG—a trinucleotide repeat that exceeds its usual range [212]. The protein function is not completely understood but has been implicated in axonal transport [213] as well as in the transcription of the brain-derived neurotrophic factor (BDNF) that is produced by cortical neurons and promotes the survival of striatal neurons in the brain [214]. This protein contains between 6–35 glutamine residues in its standard form, but in individuals affected by Huntington’s disease, it presents more than 36 glutamine residues. The mutant huntingtin protein is ubiquitously expressed, but only certain brain regions such as the hypothalamus are affected. The reason as to why only selected neurons are affected by the disease is still unknown. However, hypothalamic changes in HD impacting the regulation of metabolism, sleep, and emotional responses can be considered an early manifestation of the disease. HD is characterized by the loss of neuronal cells with typical phenotypic features in patients, such as a compromised cognitive function, personality disorder, and hyperkinetic movements over the progression of the disease [215]. BBB disruption has been observed in the in vivo model of HD. Post-mortem tissues from human patients also presented similar morphological changes [216,217]. However, the causative implication of BBB impairment in HD is not well understood. A major challenge in HD research is presented by the lack of an appropriate rodent model to reproduce the neurodegeneration and disease progression features observed in human subjects [218,219].

### 5.8. Brain Tumor

Brain tumors have been proved to negatively impact BBB integrity and permeability while promoting the formation of a blood-tumor barrier (BTB) that is highly heterogeneous and characterized by numerous distinct features, including non-uniform permeability and the active efflux of molecules [220,221]. Studies have shown that around 30% of brain tumors are metastatic and derive from lung cancer, breast cancer, and melanomas [222]. Different studies have demonstrated the breakdown of inter-endothelial TJs in gliomas and metastatic adenocarcinoma in humans [223]. The downregulated expression of TJ proteins, CLN-5, OCLN, and CLN-1, have been observed in the brain microvessels of patients with glioblastoma multiforme even though the expression of ZO-1 (a scaffold protein that cross-links and anchors the TJ strand proteins to the cell cytoskeleton), remained unaltered [217]. The explanation behind the loss of TJs in the microvessels of brain tumors remains elusive. Still, the role of VEGF and the cytokines produced by the tumor cells certainly play a significant role in the elevated BBB vascular permeability and the formation of cerebral edemas [22,224]. The latter has also been linked to the substantial upregulation of AQP4 in various brain tumors, including astrocytoma and metastatic adenocarcinoma, and has is correlated with the opening of the BBB [225]. Several in-vivo studies using brain edema models have shown that mice that lack AQP4 have better survival rates than wild-type mice. AQP4 upregulation was also reported in rat models of brain injury and ischemia [226,227]. Although the brain is highly impermeable to cancerous cells, preventing their passage into CNS, BBB disruption is likely to provide a gateway for metastatic tumor cells to enter the brain parenchyma.

It has been demonstrated that BBB/BTB structural integrity is heterogeneous with respect to metastatic lesions and tumor types [220]. For instance, the fenestration of BBB ECs differs between four molecular subtypes of medulloblastoma, which consequently affect the transcytosis of drugs across the BTB and their therapeutic efficacy [228]. Moreover, the extent of BBB properties and its function can vary among brain metastases across different subtypes of breast cancer. A higher level of GLUT1 and BCRP expression has been observed in human epidermal growth factor receptor 2 (HER2)-positive breast cancer brain metastases compared to other subtypes [229]. In addition, the heterogeneity of BTB permeability has been observed in several pre-clinical studies [220]. For instance, the center of the tumor displays a higher level of leakiness compared to the peritumoral region and the surrounding brain microenvironment. A higher distribution of liposomes containing doxorubicin within the tumor was found in the intracranial GBM8401 glioma model relative to the surrounding brain tissue [230].

### 5.9. Septic Encephalopathy

Sepsis-associated encephalopathy (SAE) is a poorly understood diffuse brain dysfunction that occurs frequently and is secondary to systemic inflammation without an overt CNS infection. SAE is frequently diagnosed in patients with a severe systemic infection (70% of cases) and those in critically ill conditions who recovered in intensive care units. The exact pathophysiology of SAE is unknown, but it appears to encompass a variety of pathogenic mechanisms, including endothelial dysfunction, reduced cerebral blood flow and oxygen extraction in the brain tissue, circulating inflammatory mediators, and cerebral edema. This cascade of events results in activation of microglial and brain endothelial cell, TJs downregulation, and increased leukocyte recruitment. Thus, the resulting neurovascular inflammation and the BBB dysfunction exacerbate SAE pathology, leading to neuronal degradation and cell death and aggravating sepsis-induced brain dysfunction [231]. BBB breakdown in septic encephalopathy was assessed through a rodent model, using multiple BBB permeability markers such as colloidal iron oxide [232], 14C amino acid [233], and 125I-albumin [234]. Increased pinocytosis, their detachment from microvessel walls, dark and shrunken neurons, and the swelling of astrocytes end-feet are pathogenic features associated with BBB disruption as presented in these animal models of septic encephalopathy [231,232]. The involvement of the adrenergic system during the inflammatory response to sepsis, including the suppression of β2 adrenoreceptor stimulation and stimulation of α1 adrenoreceptor, potentially initiate the inflammatory cascade leading to the loss of BBB integrity [235]. Further information regarding the pathogenic mechanisms of BBB dysfunction in sepsis can be found elsewhere [236,237].

### 5.10. Hepatic Encephalopathy (HE)

Hepatic encephalopathy (HE) is a complex and potentially reversible neuropsychiatric disorder resulting from acute or chronic liver failure and is characterized by drowsiness, confusion, asterixis, extrapyramidal hypertonia, convulsion, and coma [238,239]. HE results from the impaired ability of the liver to metabolize neurotoxins, particularly ammonia, leading to several psychiatric/neurological deficits [238]. Association between a disrupted BBB and HE pathogenesis has been reported in several studies. Intact BBB has been observed in HE [240]; however, positron emission tomography studies have demonstrated an increased permeability of the BBB surface area to ammonia [238]. Additionally, alterations in the expression of genes coding for endothelial nitric oxide synthase and tight junction proteins in rat brain at coma/edema stage of encephalopathy with hepatic devascularization have been reported [241]. Moreover, an increased level of neurotoxin ammonia has been reported to be associated with edema and an altered morphology of astrocytes to Alzheimer’s type II astrocytes in the basal ganglia of patients with HE. This neurotoxin has been found to cause a decreased expression level of TJ CLN-12 [242]. McClung reported that ammonia exposure causes increases in the effective pore size of the BBB under certain conditions [243]. These studies indicate the potential influence of BBB breakdown in HE pathogenesis.

### 5.11. HIV Encephalitis

The activation of astrocytes and macrophages has been associated with an infection of the human immunodeficiency virus (HIV) in the CNS. Different types of cytokines, chemokines, reactive oxygen species, and several neurotoxins released by activated astrocytes and macrophages alter neurotransmitter activities and disrupt cellular function, promoting neuronal dysfunction and leukoencephalopathy [244]. In addition to TNF-α, other factors, including NO, arachidonic acid, platelet-activating factor, and quinolinic acid, contribute to pathology. TNF-α, mainly released by HIV-infected macrophages, affects oligodendrocytes [245]. Even though the mechanism of virus entry into the CNS is still not clear, once the virus permeates the brain, it impairs the BBB integrity and facilitates a further viral load into the CNS. For instance, serum proteins were present in the brain parenchyma of patients with HIV-associated dementia [246].

Additionally, the presence of fragmented or reduced levels of TJs expression such as ZO-1 and OCLN was observed in the brains of deceased patients with HIV-1 encephalitis [247]. The extravasation of albumin and the overexpression of ICAM-1 and VCAM1 were also observed in the gp120 transgenic mice model of HIV. Circulating gp120 is thought to negatively affect the integrity of the BBB [248,249]. Similar studies have also reported the cytotoxic effect of gp120 on the ECs of the brain microvessels, which could be responsible for a higher expression of metalloproteinases and/or induced oxidative stress, thus impacting the BBB viability [250,251].

Despite treatment with antiretroviral therapy, HIV-1 associated dementia (HAD) and cognitive impairments have been observed in HIV patients [252,253]. An imbalance between the matrix metalloproteinase (MMPs) and the tissue inhibitors of metalloproteinase (TIMPs) have been identified as the prodromal factors responsible for BBB disruption and HAD pathogenesis in HIV-1 patients [254]. It has also been demonstrated that HIV-infected cells release viral proteins such as gp120, Tat, and Nef and inflammatory cytokines and chemokines, decreasing BBB integrity and viability [255].

### 5.12. Epilepsy

Several studies have reported on the association between BBB disruption and epilepsy. BBB disruption is a causative factor and/or consequence of epilepsy [256]. Studies have shown that seizure activity can cause a dysfunction of the BBB [257,258,259,260,261]. Contrastingly, ample evidence suggests that BBB disruption can result in epilepsy or aggravate the epileptic condition [262,263,264,265,266,267,268,269,270]. An opening of the BBB has been observed following a seizure, and is probably associated with acute hypertension [271,272,273]. The disruption of the BBB has also been observed after traumatic brain injury (TBI), status epilepticus (SE), and temporal lobe epilepsy (TLE). It has been reported that a widespread BBB leakage occurs within minutes of post-SE, which can last from several hours to days [262,274,275,276,277,278,279,280,281,282,283], suggesting the disruption of BBB to be a consequence of epilepsy or seizure [256].

Additionally, a leakage in BBB has been observed in epileptic patients with contrast-enhanced MRI [284,285,286]. An analysis of brain tissue collected from epileptic patients also demonstrated an elevated albumin level in the brain parenchyma, suggesting blood-to-brain extravasation of large molecules [287,288]. Furthermore, the downregulation of regional GLUT-1 and a decreased uptake and metabolism level have been observed from patient samples in different studies [287,289,290].

It has been observed that BBB disruption may be epileptogenic or may contribute to the occurs of seizures. Several studies have demonstrated that BBB permeability is most evident during the acute phase, occurring soon after SE, although it extends into the latent phase in experimental models [191,262,274,275,277]. Interestingly, intense BBB leakage has been identified during the acute and latent phases without spontaneous seizures. This result indicates that BBB disruption does not induce seizures immediately but possibly performs a significant role in epileptogenesis. Similarly, significant BBB disruption was detected directly after TBI; however, seizure activity was only observed at later stages [268,269,270]. BBB disruption partnered with osmotic shock may also result in seizures in patients [291]. Moreover, several diseases with a disrupted BBB, including stroke, TBI, infection, and inflammation, may result in epilepsy and seizures [262,292]. Patients with a GLUT-1 deficiency have also developed epilepsy, suggesting the BBB transporter as crucial to maintaining normal brain function [293,294].

Several experiments have reported the extension of BBB disruption into the chronic phase of epilepsy [191,262,274,275,277]. Quantitative measurement of BBB disruption has revealed the gradual reduction of BBB leakage during epileptogenesis in most brain regions; however, leakage can continue for weeks or months after the initial insult in the ventral brain regions in epileptic rats, although to a lesser extent than during the acute phase [262,277]. Moreover, the disruption of BBB has been observed in the resected brain tissue of patients with drug-resistant epilepsy, for which the loss of BBB integrity was detected through albumin immunohistochemistry [256]. A positive correlation has been observed between BBB leakage and the severity of a seizure in epileptic rats during the chronic phase [262]. It has been reported that the opening of the BBB by mannitol in chronic epileptic rats resulted in a stable and progressive increase in the seizure frequency [262]. Because these events do not occur instantaneously but over a more extended period, it is possible that gradual changes after BBB disruption may result in a lower seizure threshold but an increased seizure frequency. Similar results have been observed in several studies in patients with post-traumatic epilepsy. A long-lasting focal increase in BBB permeability has been reported and associated with abnormal EEG activity and a reduced cerebral blood flow [268,269,270]. Similarly, a greater level of active spiking is observed in the resected epileptogenic foci of the disrupted BBB (characterized by albumin extravasation) in comparison to albumin, which is less extravasated regions [289]. Collectively, these data indicate a relationship between the occurrence of seizures and BBB leakage, suggesting that BBB impairments can further propel epileptogenesis and the progression of (already established) epilepsy in an already diseased brain [256].

### 5.13. Schizophrenia

The disruption of the BBB has been related to forms of psychosis such as schizophrenia. A weak relationship has been observed between schizophrenia and the TJ protein, claudin 5. Around 30% of schizophrenic patients have 22q11 deletion syndrome (22q11DS) and are CLN-5 haploinsufficient. Furthermore, it has been demonstrated through in vivo studies that the adeno-associated virus-mediated inhibition of CLN-5 results in the BBB’s disruption and abnormal behavioral outcomes. Experimental in vitro and in vivo studies have revealed a dose-dependent upregulation of CLN-5 expression after treatment with antipsychotic medications. However, a discontinuous expression of CLN-5 was observed in schizophrenic patients relative to the age-matched controls when their post-mortem brain samples were analyzed [295].

Additionally, it has been reported that the overall severity of schizophrenia (OSOS) and single group negative symptoms are related to BBB disruption [296]. The correlation between deficit schizophrenia and leaky BBB has been studied. It has been concluded that deficit schizophrenia results from BBB dysfunction, secondary to the breakdown of paracellular and vascular pathways [297].

### 5.14. Meningitis

The disruption of the BBB may enhance the transportation of various compounds inside the brain by altering permeability and may cause meninges inflammation [298]. A recent study has reported that meningitic E. coli can induce PDGF-B and ICAM-1 for in vitro and in vivo models. An increment in the PDGF-B and ICAM-1 potentially contributes to breaking the BBB and neuroinflammation by downregulating TJ proteins and recruiting neutrophils or monocytes, respectively [299]. Besides, microglia perform a vital role in triggering neuroinflammation by releasing chemokines and cytokines, which ultimately results in the infiltration of white blood cells through a vascular endothelium in the BBB and thereby disrupts BBB integrity [300].

## 6. Biological Targets for Restoring BBB Viability

**Figure 3:**
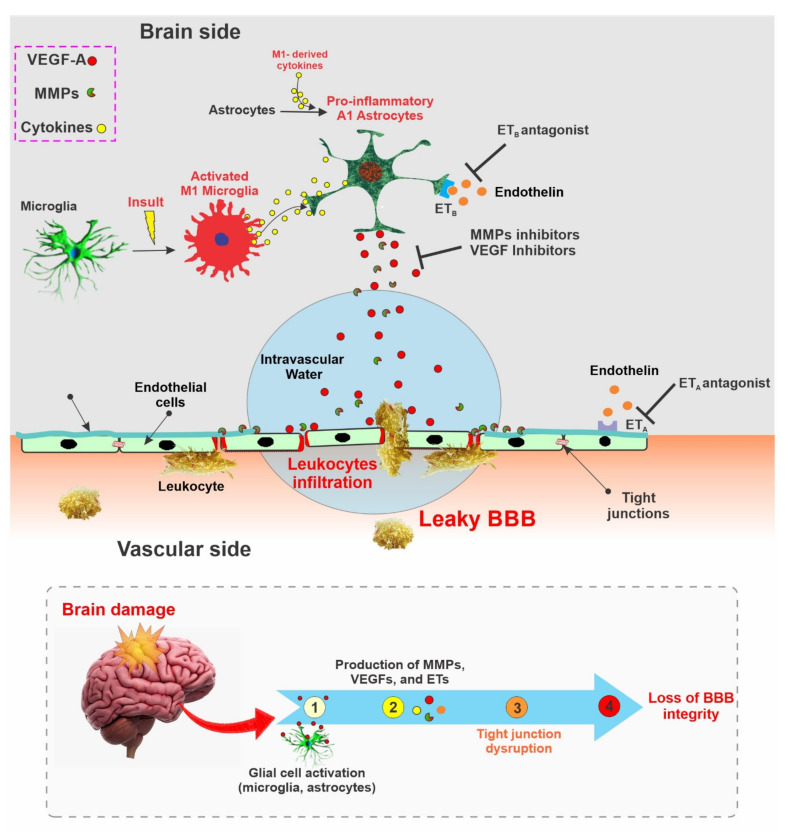
Schematic illustration summarizing the effects of brain damage on BBB integrity. The production and activation of MMPs, VEGFs, and ETs are upregulated in various brain cells following brain damage. These factors can then impair the viability and integrity of the BBB by negatively impacting the tight junctions between adjacent endothelial cells.

The neurovascular unit (NVU) consists of endothelial cells, astrocytes, and pericytes where tight junction (TJ) related proteins including CLN-5, OCLN, and ZO-1 are present in the cellular membrane of endothelial cells [301,302]. These tight junction-associated proteins ensure that the adjacent endothelial cells are tightly bound and control the movement of molecules between the intra- and the extra-vascular spaces of BBB [302]. Various kinds of brain damage can trigger different molecular pathways, which can ultimately disrupt the integrity of BBB [303,304,305]. Some of the prominent molecules related to BBB disruption include, but are not limited to, vascular endothelial growth factors (VEGFs), matrix metalloproteinases (MMPs), and endothelins (ETs) [306,307]. The interaction between microglia and astrocytes can negatively impact the integrity of the BBB and can promote neuroinflammation, which is of particular interest to this study. Microglia and astrocytes, which are part of the NVU, are activated by various brain insults within a pro-inflammatory phenotype (M1 and A1). Microglial cells, which are more sensitive to pathogens or damage, are the first to activate (into the pro-inflammatory M1 phenotype) and produce pro-inflammatory mediators, including tumor necrosis factor (TNF), interleukin 1 beta (IL-1β), and a reactive oxygen species (ROS) which then triggers the reactive astrocytes into the A1 pro-inflammatory phenotype. In this inflammatory active form, A1 astrocytes begin releasing various chemokines (which sustain a self-feedback loop of microglial activation), MMPs (which degrade the extracellular matrix), and VEGF-A, which directly impact BBB integrity by disrupting CLN-5 and OCLN TJs, thereby inducing he breakdown of BBB and immune cell infiltration [100,308] (see also Figure 3). Studies have also reported that the inhibition of these factors may restore BBB integrity (see Table 1). The potential benefits of targeting this factor for the restoration of BBB integrity is further discussed below:

### 6.1. VEGFs

VEGFs are a well-known group of angiogenic factors that include VEGF-A, B, C, and D. VEGFs promote the proliferation and migration of endothelial cells and enhance the permeability of newly formed BBB with the assistance of VEGF-specific receptors (VEGFR-1, VEGFR-2) [309,310]. Brain damage has been found to upregulate the expression of VEGF both in experimental animal studies [311,312,313] and in post-mortem patients with brain damage [314,315]. One of the mechanisms of VEGF-mediated BBB disruption is related to the downregulation of CLN-5 and OCLN expression, which is associated with VEGF upregulation in astrocytes [316]. Subsequent studies by the same group have also shown that the inhibition of astrocytic VEGF-A reduced the BBB breakdown, lymphocyte infiltration, and inflammatory damage in a mouse.

Additionally, treatment administered using cavtratin, a selective eNOS inhibitor, protected against neurologic deficits in an MS mouse model and reduced VEGF-A-induced BBB disruption [100]. Similarly, treatment administered using an anti-VEGF antibody restored the BBB, leading to improved BBB selective permeability, reduced cerebral edema, and reduced infarct volume after an ischemic stroke in mice [317]. Furthermore, treatment with the VEGF receptor 2 (VEGFR-2) inhibitor SU5416 and the knockdown of VEGFR-2 reduced BBB damage after an ischemic stroke in mice [99]. Other studies have also exhibited the potential of VEGF inhibition as a viable target for restoring the BBB [318,319].

### 6.2. Matrix Metalloproteinases

Matrix metalloproteinases (MMPs) are zinc-dependent enzymes that can disrupt BBB permeability by degrading components of the extracellular matrix (ECM). MMPs can also degrade the TJ and basal lamina proteins, ultimately disrupting the BBB and thus facilitating leukocyte infiltration, brain edema formation, and the occurrence of a hemorrhage [320]. Studies have identified the role of several MMPs, such as MMP-2, -3, -9, and -10, in BBB damage mediated through degradation of the basal lamina in brain microvessels [320,321]. In an experimental ischemic stroke and traumatic brain injury, the inhibition of MMP-2 and MMP-9 reduced BBB damage and improved the outcomes [118,322,323,324]. MMP inhibitors such as GM6001 reduce BBB disruption and brain edema [101]. Similarly, a different MMP inhibitor known as BB-1101 protected the BBB integrity and viability following an ischemic stroke [325]. The inhibition of MMPs has also been associated with a reduced expression of the intercellular adhesion molecule-1 (ICAM-1) and vascular cell adhesion molecule-1 (VCAM-1). These cell adhesion molecules are part of the immunoglobulin (Ig) superfamily and mediate the adhesion of lymphocytes, monocytes, and other immune cells to the vascular endothelium. Therefore, the downregulation of these molecules in response to MMPs’ inhibition will negatively impact inflammatory cell migration across the BBB [326]. Additionally, the inhibition of MMP2/9 with SB-3CT after schizophrenia in mice promoted BBB recovery and accelerated their neurological recovery [118]. Moreover, a tetracycline antibiotic, minocycline, has also been demonstrated to inhibit MMP enzyme and MMP production [327,328] which may ultimately reduce BBB disruption.

### 6.3. Endothelins

Endothelins (ETs) are endogenous vasoconstrictive proteins and are responsible for several CNS physiological and pathological consequences. The overexpression of ET-1 has been associated with BBB breakdown and cognitive dysfunction after an ischemic stroke in mice [329,330]. Furthermore, the upregulation of ET-1 promoted the expression of MMP2 and downregulated that of the inter endothelial TJ OCLN after an ischemic stroke [329]. Similarly, increased levels of ET-1 after a hemorrhagic stroke was associated with BBB disruption and brain edema formation [331]. Endothelin receptors, ET_A_, and ET_B_, have also been associated with ET-mediated BBB damage, and ET_A_ antagonist, S-0139, was also found to reduce post-stroke increased BBB permeability and edema formation in mice [102]. Similarly, an anti-ET_B_ known as BQ788 was found to significantly improve BBB integrity in an experimental animal model of epilepsy by inhibiting MMP-9 and ZO-1 degradation [103,104].

### 6.4. Adherens Junctions

Furthermore, changes in adherens junction (ADs) proteins expression have been observed under different pathological conditions and associated with BBB disruption. For instance, decreased cadherin expression or loss of cadherin has been observed in stroke, TBI, and brain tumors, with BBB integrity loss. In contrast, the restoration of their expression resulted in tight junction repair and disease regression [332]. However, the options that are currently available to restore ADs expression are limited. The use of CD5-2, a miR-27a/VE blocker, has been shown to increase VE-cadherin expression [105]. In fact, CD5-2 significantly improved BBB integrity and reduced cerebral cavernous malformation lesions in mice [333]. Additionally, a bioactive sphingolipid known as sphingosine-1-phosphate (S1P) has been shown to preserve BBB integrity by stabilizing the cadherin at the endothelial cell-cell contact regions [106]. Due to the functional link between adherens junctions and tight junctions, the modulation of cadherin may impact the formation of inter-endothelial tight junctions.

### 6.5. Tight Junctions

A decreased expression of tight junction proteins such as CLN-5 and ZO-1 has been observed in different neurological disease conditions. In mouse models of depression, chronic treatment with anti-depressants resulted in an increased CLN-5 expression [334]. An experimental antisense oligonucleotide for miR-501-3p prevented ZO-1 downregulation and cognition impairments in mice [107]. Similarly, a treatment with MS-275 (Entinostat), a histone deacetylase 1 (HDAC1) inhibitor, improved the BBB claudin-5 expression and reduced depression-like syndrome in a mice model of stress [108]. Likewise, melatonin treatment has also been associated with the mitigation of tight junction dysfunction through AMP-activated protein kinase [109].

Additionally, studies have shown the downregulation of tight junction proteins (CLN-5, OCLN, and ZO-2) after traumatic brain injury in mice [335]. Recent experimental data have also shown that Nrf2 enhancers such as metformin and sulforaphane can mitigate the post-injury reduction of tight junction proteins [336,337]. Similarly, blast-induced traumatic brain injury in mice reduced the BBB expression of tight junction proteins, and the phenomenon was reversed with a post-injury administration of dexamethasone [338]. Another compound, 4-hydroxy-2,2,6,6-tetramethylpiperidine-N-oxyl (TEMPOL), a membrane-permeable radical scavenger, has also been demonstrated to improve BBB disruption by enhancing the expression of tight junction proteins after ischemic injury in a rat model of transient focal ischemia and splanchnic artery occlusion and reperfusion [339,340].

### 6.6. Endothelium

The endothelium is the thin layer of specialized endothelial cells that lines the inner luminal surface of blood vessels, and it is the innermost part of BBB. This layer of endothelial cells controls the vascular tone, blood fluidity, and extravasation of blood components within the brain parenchyma [3]. An injury or the dysfunction of this endothelium of BBB results in neurovascular inflammation, oxidative stress, thrombosis, and ischemia; therefore, this represents an important therapeutic target for several neurovascular disorders which affect BBB [341]. The study has shown the protective role of superoxide dismutase (SOD) conjugated with antibody (Ab/SOD) in managing acute vascular inflammation [342]. Similarly, ICAM-1 targeted catalase resulted in a marked reduction in oxidative stress, restored BBB integrity, and improved neurological function [343]. Enlimomab, a murine antibody for human ICAM-1, presented a significant therapeutic effect in repairing BBB after ischemic stroke in animal studies (101). Additionally, in a rat model of middle cerebral artery occlusion (MCAO) and a model of an ischemic stroke, the systemic administration of SOD1-cl-nanozymes reduced brain tissue injury along with sensory motor function recovery in rats [344].

### 6.7. Cytokines

One of the prominent characteristics of compromised BBB is the upregulation of inflammatory cytokines, such as TNF-β, IL-1β, TNF-α, and IL-6 [334]. Inhibiting these inflammatory cytokines has been shown to protect BBB integrity and ameliorate CNS disorders [345,346,347]. Treatment with the TNF-α inhibitor etanercept improved BBB integrity in a mouse model of depression [110]. Similarly, the use of anti-IL-6 antibodies improved BBB integrity in ovine fetuses [111]. Additionally, natalizumab, a humanized monoclonal antibody against the cell adhesion molecule α4-integrin, has been able to inhibit BBB endothelial inflammation by blocking the interaction between α4 integrin on white blood cells that are involved in inflammation and the VCAM-1 expressed on the vascular endothelium, thereby preventing white blood cells from entering the brain and spinal cord tissue [112,113].

### 6.8. Oxidative Stress

A critical class of intracellular signaling molecules known as reactive oxygen species (ROS), if accumulated in excess, can cause oxidative stress and cell damage, eventually leading to cell death. Increased production of ROS and oxidative stress have been associated with several neurological disorders, such as Alzheimer’s disease, Parkinson’s disease, Huntington’s disease, multiple sclerosis, and stroke [348,349]. The activation of two oxidative stress-inducing enzymes, NADPH oxidase 4 (NOX4) and NOX5 promote BBB breakdown, leading to the infiltration of inflammatory cells in the brain parenchyma [114,115]. In a cerebral ischemic mice stroke model, the selective inhibition of NOX4 by GKT136901 (a NOX-1/4 inhibitor with potential application in diabetic nephropathy, stroke, or neurodegeneration) and NOX5 by ML090 (a pan-NOX inhibitor) improved BBB integrity and reduced infarct volume [114,115]. Additionally, the nuclear factor erythroid 2 (NFE2)-related factor 2 (Nrf2) is an emerging regulatory factor that participates in the modulation of cellular oxidative stress through the transcription of an array of antioxidant and detoxifying genes [350]. A reduced level of Nrf2 has been associated with increased susceptibility to brain injury due to its effect on BBB integrity [351], given that this nuclear factor has also been shown to regulate the expression of BBB endothelial tight and adherens junction proteins such as ZO-1 and OCLN [352]. Consequently, compounds such as metformin and sulforaphane, which enhance Nrf2 expression and activity, have been shown to effectively protect and/or restore BBB integrity after brain injuries [336,337].

### 6.9. Actin-Myosin Cytoskeleton

Phosphorylation of the myosin light chain promotes the contraction of the actin-myosin cytoskeleton, leading to an increased cytoskeleton tension, junction protein disorganization, and the widening of paracellular space, ultimately impacting the BBB [127]. From a mechanistic perspective, both phosphorylation and tight junction protein internalization are mediated by the activation of RhoA/Rho-associated proteins kinase (ROCK). Phosphorylation of tight junction proteins OCLN and CLN-5 by ROCKs promotes the migration of monocytes across the BBB [353]. Additionally, ROCKs activation in the capillaries of a mice model of AD led to BBB disruption and increased microvascular permeability [354]. Hence, the inhibition of ROCK1/ROCK2 has been proven to counteract BBB disruption resulting from brain injuries, such as cerebral ischemia, experimental autoimmune encephalomyelitis, and intracerebral hemorrhages [355]. Even though there are selective pharmacological inhibitors for ROCK1/ROCK2 isoforms, non-isoform specific ROCKs inhibitors, fasudil and Y-27632 are the most widely explored. Treatment using fasudil reduced the disruption of BBB and cavernous cerebral malformation in a murine model of an ischemic stroke [116,117]. Similarly, the inhibition of ROCK with Y-27632 or an ischemic stroke significantly reduced the cerebral lesion and edema volumes and improved the functional outcomes [356]. In the same study, the treatment of brain microvascular endothelial cells exposed to oxygen-glucose deprivation (OGD), using Y-27632 prevented the loss of intercellular junctions. Additionally, Slx-2119 (KD025) and SR3677, ROCK2-specific inhibitors, have also been shown to protect the cerebrovascular integrity in cerebral ischemia and reduce the production of amyloid-β in a mouse model of Alzheimer’s disease, respectively [357,358].

## 7. Conclusions

**Figure 4:**
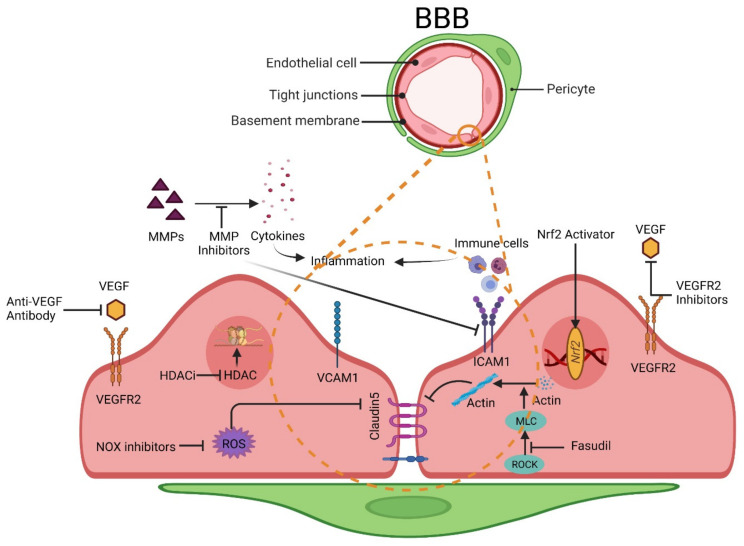
Structure of blood-brain barrier (BBB) and potential therapeutic targets to restore the BBB viability. Targeting angiogenesis, oxidative stress, cytoskeleton reorganization, and inflammation can effectively protect and potentially restore the viability of the BBB. This includes MMPs and angiogenesis inhibitors (e.g., antibodies targeting VEGF/VEGFR), oxidative stress inhibitors/reducers (such as NOX inhibitors and Nrf2 enhancers) that are promoters of cytoskeleton reorganization (e.g., ROCK inhibitors). Blocking inflammation by targeting immune cells can prevent their recruitment, thus protecting the BBB.

The BBB is a vital cellular and biological barrier that maintains the CNS microenvironment’s homeostasis by controlling the movement of molecules into and out of the brain parenchyma. This barrier acts as a gatekeeper so as to protect the brain from toxins, chemicals, inflammation, and pathogens. Therefore, a disruption of the BBB can lead to the onset and progression of several cerebrovascular and neurological diseases, which can be of a chronic or acute type [359]. In this review, we have summarized the role and functions of the BBB, the neurological disorders that are directly or indirectly associated with BBB disruption, and discussed potential therapeutic targets for restoring the impaired BBB. Studies have shown the involvement of different signaling pathways, such as VEGF, TNF-α, MMP and ET, in promoting the disruption of the BBB. Various experimental and clinical studies have revealed that some small molecular factors bear the potential of becoming viable therapeutic targets of intervention (see Figure 4). Unfortunately, clinical treatments that are capable of effectively restoring the disrupted BBB do not yet exist.

Even though substantial progress has been made in identifying many of the putative mechanisms and key factors involved in BBB disruption, further well-controlled pre-clinical and clinical studies are required to validate these pathogenic mechanisms. Furthermore, it is critically important to assess the pros and cons of modulating the activity of these molecular targets to promote the protection and/or restoration of BBB functions and integrity.

Understanding the relationship between BBB disruption and the subsequent brain pathophysiological cues is crucial for developing more effective and specific therapeutic strategies. Today, there is a major lacuna in our understanding of the underlying mechanisms linking BBB dysfunction and neurological disorders. In several instances, whether the impairment of the BBB is a direct causative factor of the CNS disorder or, instead, a result of the neurological disease, which can later negatively impact the outcome, is also unclear. However, regardless of its potential role as a prodromal factor for the onset of CNS disorders or a collateral casualty, BBB disruption has been established as a key player in worsening the disease’s outcome. Hence, combinatorial therapies targeting both the BBB and the brain parenchyma are more desirable than treatments targeting each of them as singular issues.

Moreover, due to the inherited heterogeneity of the brain structure and function, it is also important to consider region-specific BBB damage and subsequent therapeutic targets. Novel technologies are becoming available to assist in designing and delivering more effective and selective therapeutic strategies. These advanced techniques, like transcriptomic and proteomic analysis, single-cell RNA sequencing, and other advanced technologies, can help to elucidate the mechanistic differences of BBB damage as related to specific regional structures of the brain. Such an approach would assist in providing the necessary assistance for the development of more effective treatments.

## Figures and Tables

**Table 1:** Blood-Brain Barrier disruption associated with different disease conditions and potential therapeutic targets.

Pathophysiology of BBB Disruption	Associated Diseases	Therapeutic Targets	Ref.
Upregulation of VEGF and Activation of VEGFR2	ALS, AD, PD, epilepsy, ischemic stroke	anti-VEGF antibody and VEGFR 2 inhibitor (e.g., SU5416)	[99]
eNOS activation	Stroke and TBI	selective eNOS inhibitor (e.g., cavtratin)	[100]
Upregulation of MMPs	Schizophrenia, Stroke, and TBI	Inhibition of MMPs (e.g., GM6001)	[101]
Activation of endothelin receptors, ET_A_, ET_B_	Stroke and epilepsy	Inhibition of ET_A_ and ET_B_ (e.g., S-0139, BQ788)	[102,103,104]
Downregulation of VE-Cadherine	MS, Stroke	Inhibition of miR-27a/VE by CD5-2; antibody against ICAM (Enlimomab)	[105]
Disorganization of adherens junctions	MS, Stroke	Stabilize the adherens junctions using sphingosine-1-phosphate (S1P)	[106]
Reduced expression of TJ proteins	Depression, Stroke, Stress,	Induction of TJs protein expression. (e.g., antisense oligonucleotide for miR-501-3p, HDAC1 inhibitor, MS-275 -Entinostat)	[107,108]
Imbalance of AMP-activated protein kinase pathway	Stroke	Activation of AMP-activated protein kinase pathway by melatonin	[109]
Activation or upregulation of inflammatory cytokines e.g., TNF-β, IL-1β, TNF-α and IL-6	Stroke, TBI, Cognitive impairments, Seizures, MS	Inhibition of inflammatory cytokines (e.g., etanercept, anti-IL-6 antibody, and natalizumab)	[110,111,112,113]
Oxidative stress induction by activation of NOX4 and NOX5	Stroke	selective inhibition of NOX4 or NOX5 (GKT136901 and ML090)	[114,115]
Contraction of actin-myosin cytoskeleton via myosin light chain Phosphorylation	Stroke	Inhibition of RhoA/Rho-associated proteins kinase (ROCK). e.g., fasudil	[116,117]
Upregulation of MMPs	Stroke, TBI and Schizophrenia	Inhibition of MMP2/9 (e.g., SB-3CT)	[118]

## Data Availability

Not applicable.

## Abbreviations

AD	Alzheimer’s Disease
ALS	Amielotropic Lateral Sclerosis
BBB	Blood-Brain Barrier
BMECs	Human Brain Microvascular Endothelial Cells
CLN-5	Claudin-5
CNS	Central Nervous System
GLUT-1	Glucose Transporter
HDAC	Histone Deacetylases
HDAC i	HDAC inhibitor
HIV	Human Immunodeficiency Virus
ICAM1	Intercellular Adhesion Molecule 1
MLC	Myosin Light Chain
MMPs	Matrix Metalloproteases
MRP	Multidrug Resistance-Associated Protein
MS	Multiple Sclerosis
NOX	NADPH Oxidase
Nrf2	Nuclear Factor Erythroid 2 (NFE2)-Related factor 2)
NVU	Neurovascular Unit
OCLN	Occludin
PD	Parkinson’s Disease
P-gp	P-glycoprotein
VEGF	Vascular Endothelial Growth Factor
ROCK	RhoA/Rho-Associated Proteins Kinase
ROS	Reactive Oxygen Species
VCAM1	Vascular Cell Adhesion Protein 1
VEGFR2	Vascular Endothelial Growth Factor Receptor-2
TJs	Tight Junctions
ZO-1	Zona Occludens-1
3D	3 Dimensional
